# Dieulafoy disease of the bronchus involving bilateral arteries: A case report and literature review

**DOI:** 10.1097/MD.0000000000017798

**Published:** 2019-11-01

**Authors:** Pan Tang, Tingting Wu, Chaofen Li, Chengna Lv, Jing Huang, Zaichun Deng, Qunli Ding

**Affiliations:** a Department of Pulmonary and Critical Care Medicine, The Affiliated Hospital of Medical College, Ningbo University; b Department of Chemical Biology and Clinical Laboratory, Ningbo Ninth Hospital,; c Department of Pharmacy, The Affiliated Hospital of Medical College, Ningbo University, Ningbo, China.

**Keywords:** bronchoscopy, computed tomography angiography of bronchial artery, Dieulafoy disease of the bronchus, Hemoptysis, vascular deformity

## Abstract

**Rationale:**

Dieulafoy disease of the bronchus is a rare vascular deformity. To the best of our knowledge, reports of these involving both lung vascular are hitherto absent.

**Patient concerns:**

A 67-year-old male was admitted to our department due to agnogenic hemoptysis.

**Diagnoses:**

Bronchoscopy was performed and some smooth, pulsatile nodular lesions were found in the middle and lower lobes, Computed tomography angiography of the bronchial artery confirmed a left bronchial artery arising from the aortic arch at T4 level, and both bronchial arteries were dilated and tortuous.

**Interventions:**

Bronchial artery embolization was performed successfully.

**Outcomes:**

The patient was discharged with no hemoptysis. In addition, patient is under follow-up until today without any further incidents.

**Lessons:**

This case reminds us that Dieulafoy disease of the bronchus could be a potential etiology for unexplained hemoptysis. The clinician should be aware of this disease when bronchoscopy revealed multiple some smooth, pulsatile nodular lesions, thereafter, bronchoscope biopsy should be avoided, as it could lead to fatal hemoptysis.

## 1 Introduction

Dieulafoy disease is a rare vascular anomaly characterized by dilated, tortuous arteries in the submucosa. It was observed to occur commonly in the gastrointestinal tract and was described for the first time by Dieulafoy in 1898. Dieulafoy disease of the bronchus is rarely seen, which could be presented with recurrent and unexplained massive hemoptysis or with asymptomatic. To our knowledge, 29 cases have been reported since it was first reported by Sweets in 1995,^[1]^ but involving bilateral vascular lesions is hitherto absent. Here, we present the case of a 67-year-old male who was diagnosed to have Dieulafoy disease of the bronchus, computed tomography angiography of the bronchial artery confirmed a left bronchial artery arising from the aortic arch at T4 level, and both bronchial arteries were dilated and tortuous.

## 2 Case report

A 67-year-old nonsmoking man was admitted with productive cough and hemoptysis. He had been hospitalized 8 months earlier because of the same symptom and coughed up approximately 10 ml of fresh blood. After cessation of bleeding, there was no hemoptysis during the period before this hospitalization. One day before the current admission to our hospital, he developed recurrent hemoptysis and coughed up fresh blood (about 100 ml) without any clots.

Physical examination of the head, neck, chest, and abdomen was within normal limits. Laboratory tests including white blood cell count, hemoglobin level, platelet count, hematocrit, prothrombin time, partial thromboplastin time (PT), and international normalized ratio (INR) did not reveal any obvious abnormal findings.

**Figure 1:**
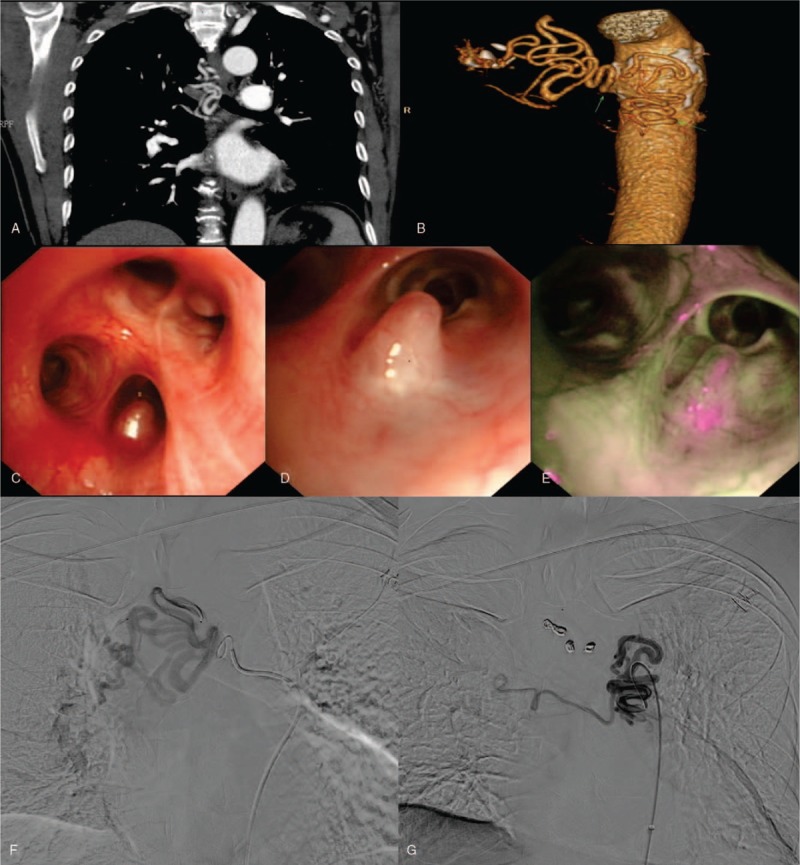
(A, B) CT angiography shows a left bronchial artery arising from the aortic arch at T4 level, from which a branch supplied the pulmonary circulation and the bronchial arteries on both sides were dilated and tortuous; (C, D) Bronchoscopy of the right lung shows 2 smooth and pulsatile nodular lesions located at the opening of the apical and posterior segments of the superior lobe, and fluorescent staining revealed green fluorescence (E); (F, G) Angiography of the bronchial arteries shows that both arteries were dilated, tortuous, and deformed.

Computed tomography angiography showed a left bronchial artery arising from the aortic arch at T4 level, from which a branch supplied the pulmonary circulation, and both bronchial arteries were dilated and tortuous (Fig. 1A, B).

Bronchoscopy was performed and some smooth, pulsatile nodular lesions were found in the middle and lower lobes; 2 cone-shaped endobronchial protrusions covered by a smooth mucosa were noticed in the right upper lobe (Fig. 1C, D), and fluorescent staining revealed green fluorescence (Fig. 1E). We did not perform a biopsy because we suspected that vascular lesions were present in the bronchial tree.

Then, bronchial angiography was performed and it showed bilateral bronchial arteries were dilated and tortuous (Fig. 1F, G). Then, bronchial artery embolization was performed successfully. The patient was discharged with no hemoptysis. In addition, patient is under follow-up until today without any further incidents. Informed consent was obtained from the patient for publication of this case report and accompanying images.

## 3 Discussion

Massive hemoptysis is a common critical disease in respiratory medicine. There are many causes of hemoptysis, such as bronchiectasis, tuberculosis, or neoplasm. Dieulafoy disease of the bronchus is a rare clinical entity.

Dieulafoy disease is a rare vascular anomaly characterized by dilated, tortuous arteries in the submucosa.^[2]^ It appears to occur throughout the gastrointestinal tract. Since Dieulafoy disease of the bronchus was first reported by Sweerts et al in 1995,^[1]^ multiple similar cases have been reported in the literature.^[1–23]^ However, the pathogenesis of this vascular anomaly is unclear.^[3]^ Herein, we present a case of Dieulafoy disease of the bronchus and then we identified 23 published reports with a total of 29 cases and summarized the clinical features of all the cases in Table 1.

**Table 1:**
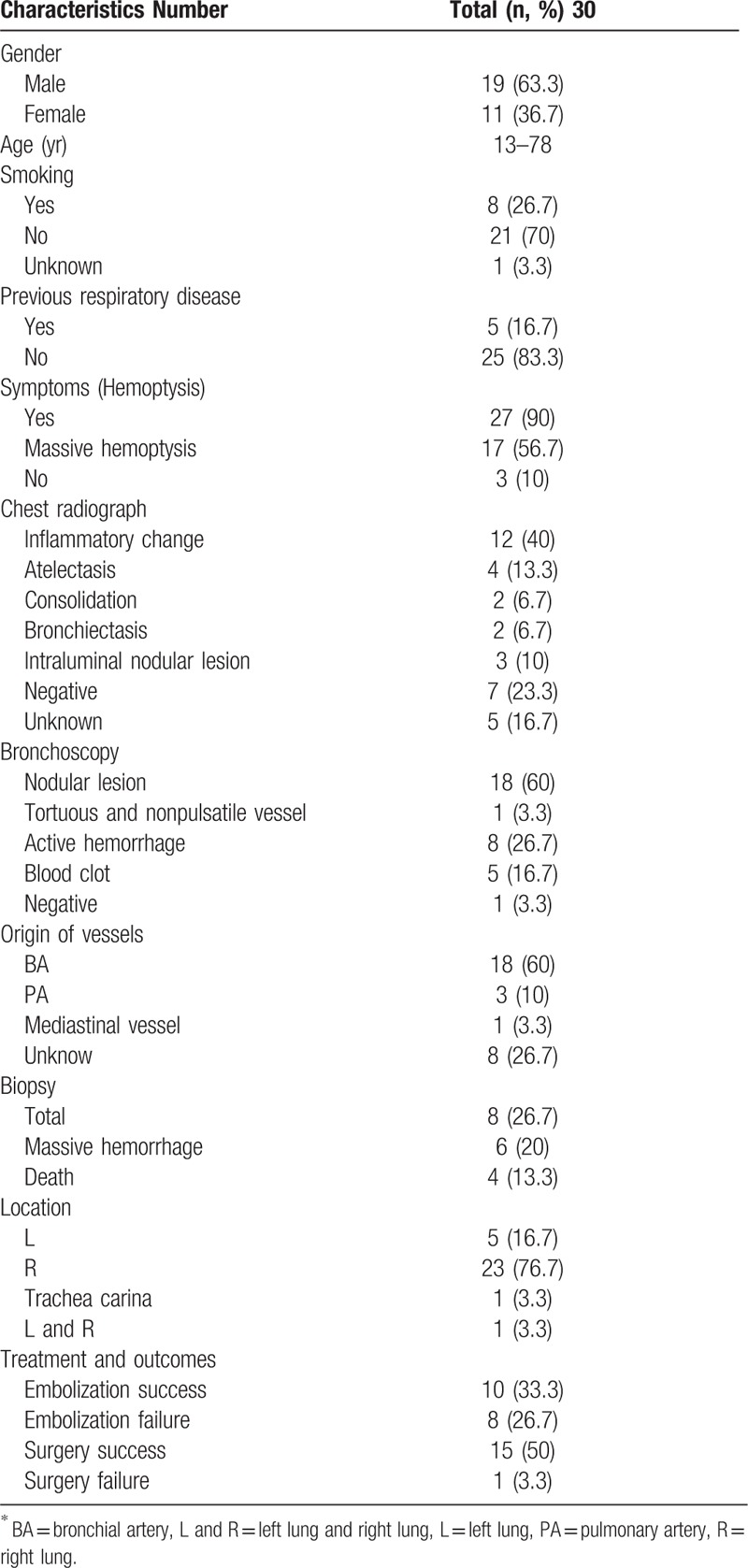
Clinical manifestations of Dieulafoy disease of the bronchus^∗^^[1–23]^.

Studies show that this condition is associated with bronchial pulmonary congenital dysplasia, chronic airway inflammation, or injury.^[4]^ Most patients had no history of smoking (21/30) or existing respiratory diseases (25/30). Dieulafoy disease of the bronchus should be suspected if a patient is suffering from recurrent and unexplained episodes of hemoptysis. However, the clinical manifestations are non-typical, comprising productive cough, chest pain, recurrent pneumonia, or exacerbation of bronchiectasis.

In most cases, only inflammatory change was observed, with atelectasis or a non-significant abnormality on routine imaging examination, either on a chest X-ray image or chest CT scan. Only 3 cases presented with an intraluminal nodular lesion and 2 cases showed consolidation on chest CT scan. However, CT angiography is a useful diagnostic tool to detect and locate the origin of abnormal arteries and bleeding.^[5]^ In our study, we found that CT angiography have been performed on only 6 cases of 30 patients which revealed bronchial artery malformation. The examination revealed the presence of a dilated, tortuous bronchial artery and bronchial-pulmonary artery fistula. According to the literature, the dysplastic artery appears in the right (23/30) lung more than it does in the left side (5/30), and bilateral vascular lesions are rarer (1/30).^[1]^ The incidence of lesions seems higher on the right side, but in our case, the lesions were bilateral vascular involvement. According to the observations from the pathological examination of dissection specimens, these dilated or deformed arteries pass the bronchial wall and travel through the submucosa.^[4]^ Besides, abnormal vessels arise more from the systemic than the pulmonary circulation. At present, bronchial arteriography is a useful method for making a definite diagnosis.

Through bronchoscopy, mucosal abnormalities are expressed as small (about 2–7 mm in diameter), non-pulsatile, and smooth nodular lesions with a white cap (18/30), with active hemorrhage (8/30) and blood clot in the bronchus (5/30). Nevertheless, the diagnosis depends on pathological examination; however, biopsy should be considered carefully because of the risk of fatal hemorrhage.^[6]^ Among the 30 cases in this study, 8 patients underwent a biopsy, 6 cases presented massive hemorrhage and 4 patients died. Therefore, we do not recommend bronchoscopic biopsy once the disease is suspected.

When bronchoscopy is performed in a patient presenting with hemoptysis, it is important to identify the characteristics of the intraluminal nodular lesions, thus reducing the risk of fatal hemorrhage. With the use of a conventional white light bronchoscope alone, up to 29% of the vascular anomalies and dysplastic mucosal and dysplastic mucosal lesions may not be detected.^[24,26]^ Narrow band imaging (NBI) can aid in visualizing the superficial mucosa to detect vascular lesions by using 2 different ranges of wavelengths: 390 to 445 nm and 530 to 550 nm.^[26]^ Vascular lesions in the submucosa appear blue-green under NBI; the color blue indicates that the vessel is at a shallow location, while green indicates that the location is at a deeper location. Endobronchial ultrasound scan is also a useful tool to detect vascular abnormalities which are usually hypoechoic or anechoic with hyperechoic or echogenic walls.^[24,25]^ Combining these 2 technologies can help to define the property of intraluminal nodular lesions.

Up till now, selective bronchial artery embolization has been an effective and safe procedure for controlling hemoptysis. This procedure offers an alternative method for patients who cannot undergo lobectomy owing to compromised general health status or simply do not want to undergo surgery and demonstrates a good curative effect (10/18). Despite this, 8 cases presented with recurrent hemoptysis, which may have been associated with malformed vessels originating from the pulmonary circulation rather than systemic circulation. In addition, revascularization, neovascularization, and drop of the embolus dropping could also have caused failed embolization.^[5]^

If the malformed vessels are originating from the pulmonary circulation, embolization is often ineffective, and lobectomy can be an alternative option for cure. Lobectomy should be considered when the patient develops massive hemoptysis or embolization is unsatisfactory.

To the best of our knowledge, reports of these involving both lung vascular are hitherto absent. We present the first case report of recurrent hemoptysis resulting from dilated and tortuous bilateral bronchial arteries causing Dieulafoy disease. Our patient had a short-term history of slight hemoptysis. Moreover, the time of his first episode compared to others was relatively late. The bronchial angiography and CT angiography revealed the same features, that is, dilated and tortuous bilateral bronchial arteries; however, this is an infrequent situation within clinical practice. Then, the patient showed satisfactory curative effect through bronchial artery embolization.

In summary, this case we reported and those reviewed support that Dieulafoy disease of the bronchus should be considered if a patient presents with recurrent and unexplained hemoptysis. Combining narrow band imaging and endobronchial ultrasound scan can help to identify the characteristics of an intraluminal nodular lesions before biopsy, thus reducing the risk of fatal hemorrhage. Bronchial arteriography and selective bronchial artery embolization should be performed in time to reduce the risk of life threatening hemoptysis. Lobectomy could be considered as a radical intervention.

## Author contributions

**Conceptualization:** Zaichun Deng.

**Data curation:** Pan Tang, Chengna Lv.

**Formal analysis:** Chaofen Li.

**Funding acquisition:** Qunli Ding.

**Investigation:** Tingting Wu.

**Methodology:** Jing Huang.

**Project administration:** Qunli Ding.

**Writing – original draft:** Pan Tang.

Qunli Ding orcid: 0000-0002-6181-4548.

## References

[1] Sweerts M Nicholson AG Goldstraw P Dieulafoy's disease of the bronchus. Thorax 1995;50:697–8. 7638820 10.1136/thx.50.6.697 PMC1021280

[2] Smith B Hart D Alam N Dieulafoy's disease of the bronchus: a rare cause of massive hemoptysis. Respirol Case Rep 2014;2:55–6. 25473566 10.1002/rcr2.47 PMC4184505

[3] Stoopen E Baquera-Heredia J Cortes D Dieulafory's disease of the bronchus in association with a paravertebral neurilemoma. Chest 2001;119:292–4. 11157620 10.1378/chest.119.1.292

[4] Wang YF Zeng YM Dieulafoy′s disease of bronchus: a case report and review of liferature. Int J Respir 2015;35:1719–22.

[5] Fang Y Wu QC Wang B Dieulafoy's disease of the bronchus: report of a case and review of the literature. J Cardiothorac Surg 2014;19:191–5. 10.1186/s13019-014-0191-8 PMC4263116 25438694

[6] Van der Werf TS Timmer A Zijlstra JG Fatal haemorrhage from Dieulafoy's disease of the bronchus. Thorax 1999;54:184–5. 10325926 10.1136/thx.54.2.184 PMC1745428

[7] Maxeiner H Lethal hemoptysis caused by biopsy injury of an abnormal bronchial artery. Chest 2001;119:1612–5. 11348980 10.1378/chest.119.5.1612

[8] Bhatia P Hendy MS Li-Kam-Wa E Recurrent embolotherapy in Dieulafoy's disease of the bronchus. Can Respir J 2003;10:331–3. 14530826 10.1155/2003/729714

[9] Pomplun S Sheaff MT Dieulafoy's disease of the bronchus: an uncommon entity. Histopathology 2005;46:598–9. 15842649 10.1111/j.1365-2559.2005.02024.x

[10] Loschhorn C Nierhoff N Mayer R Dieulafoy's disease of the lung: a potential disaster for the bronchoscopist. Respiration 2006;73:562–5. 16141709 10.1159/000088059

[11] Kolb T Gilbert C Fishman E Dieulafoy's disease of the bronchus. Am J Respir Crit Care Med 2012;186:1191. 23204378 10.1164/rccm.201206-1016IM PMC7051586

[12] Barisione EE Ferretti GG Ravera SS Dieulafoy's disease of the bronchus: a possible mistake. Multidiscip Respir Med 2012;7:40–2. 23137343 10.1186/2049-6958-7-40 PMC3517463

[13] D’ Souza F Sharma R Dieulafoy's disease of the bronchus. Pathology 2010;42:683–4. 21080881 10.3109/00313025.2010.523687

[14] Wadji MB Farahzadi A Dieulafoy's disease of the bronchial tree: a case report. Sao Paulo Med J 2017;135:396–400. 28562735 10.1590/1516-3180.2016.0258191116 PMC10015996

[15] Venus A Uthamalingam P Sitaram B Dieulafoy's disease of the lung: a report of three cases with review of pathological aspects. Indian J Thorac Cardiovasc Surg 2016;32:217–20.

[16] Yang DR Rong CH Gu J Dieulafoy disease of the trachea with recurrent episodes of massive hemoptysis: a case report. Medicine 2017;96:e5855. 28151860 10.1097/MD.0000000000005855 PMC5293423

[17] Gharagozloo F Rennert D Margolis M Dieulafoy lesion of the bronchus: review of the literature and report of the 13th case. J Bronchol 2008;15:38–40.

[18] Xie BS Chen YS Lin MF Dieulafoy's disease of the bronchus: a case report and review of the literature. Chin J Tuberc Respir Dis 2006;29:801–3. 17327080

[19] Wan W Xia Y Huang HD Dieulafoy's disease of the bronchus: a case report and review of liferature. Int J Respir 2011;31:919–22.

[20] Yang RH Li JF Liu J Dieulafoy disease of the bronchus: 3 cases report with literature review. Chin J Tuberc Respir Dis 2013;36:577–80. 24252733

[21] Bonnefoy V Garnier M Tavolaro S Bronchial Dieulafoy's disease: visualization of embolization particles in bronchial aspirate. Am J Respir Crit Care Med 2018;198:954–5. 29877731 10.1164/rccm.201711-2184IM

[22] Foo AZX Hsu AAL Dieulafoy's disease with mediastinal arteriovenous malformation. Thorax 2018;73:691–2. 10.1136/thoraxjnl-2017-211243 29353256

[23] Wang F Kuang TG Wang JF A rare cause of recurrent fatal hemoptysis: Dieulafoy's disease of the bronchus. Chin Med J 2018;131:2758–9. 30425206 10.4103/0366-6999.245279 PMC6247593

[24] Ganganah O Guo SL Chiniah M Endobronchial ultrasound and bronchial artery embolization for Dieulafoy's disease of the bronchus in a teenager: a case report. Respir Med Case Rep 2015;16:20–3. 26744645 10.1016/j.rmcr.2015.04.007 PMC4681900

[25] Gurioli C Casoni GL Gurioli C Endobronchial ultrasound in Dieulafoy's disease of the bronchus: an additional application of EBUS. Monaldi Arch Chest Dis 2010;73:166–8. 21434565 10.4081/monaldi.2010.287

[26] Weisberg MD Garza EG Tabor MH The use of narrow band imaging in patients with benign disease: hereditary hemorrhagic telangiectasia. J Bronchol Intervent Pulmonol 2011;18:352–4. 10.1097/LBR.0b013e3182342346 23208632

